# Comparison of Risks Factors for Unplanned ICU Transfer after ED Admission in Patients with Infections and Those without Infections

**DOI:** 10.1155/2014/102929

**Published:** 2014-01-02

**Authors:** Jeffrey Che-Hung Tsai, Ching-Wan Cheng, Shao-Jen Weng, Chin-Yin Huang, David Hung-Tsang Yen, Hsiu-Ling Chen

**Affiliations:** ^1^Department of Emergency Medicine, China Medical University Hospital, No. 2 Yude Road, North District, Taichung City 404, Taiwan; ^2^School of Medicine, College of Medicine, China Medical University, Taichung 404, Taiwan; ^3^Department of Industrial Engineering & Enterprise Information, Tunghai University, Taichung 407, Taiwan; ^4^Department of Emergency Medicine, Cheng-Ching General Hospital, Taichung 407, Taiwan; ^5^Program for Health Administration, Tunghai University, Taichung 407, Taiwan; ^6^Institute of Emergency and Critical Care Medicine, College of Medicine, National Yang-Ming University, Taipei 112, Taiwan

## Abstract

*Background*. The objectives of this study were to compare the risk factors for unplanned intensive care unit (ICU) transfer after emergency department (ED) admission in patients with infections and those without infections and to explore the feasibility of using risk stratification tools for sepsis to derive a prediction system for such unplanned transfer. *Methods*. The ICU transfer group included 313 patients, while the control group included 736 patients randomly selected from those who were not transferred to the ICU. Candidate variables were analyzed for association with unplanned ICU transfer in the 1049 study patients. *Results*. Twenty-four variables were associated with unplanned ICU transfer. Sixteen (66.7%) of these variables displayed association in patients with infections and those without infections. These common risk factors included specific comorbidities, physiological responses, organ dysfunctions, and other serious symptoms and signs. Several common risk factors were statistically independent. *Conclusions*. The risk factors for unplanned ICU transfer in patients with infections were comparable to those in patients without infections. The risk factors for unplanned ICU transfer included variables from multiple dimensions that could be organized according to the PIRO (predisposition, insult/infection, physiological response, and organ dysfunction) model, providing the basis for the development of a predictive system.

## 1. Introduction

The emergency department (ED) is an important source of hospital inpatients, especially those with critical problems. The condition of some patients may deteriorate after transfer from the ED to a general ward, necessitating an unplanned transfer to the intensive care unit (ICU). Patients with an unplanned ICU transfer after an ED admission have a higher rate of mortality compared to that for patients who are admitted directly to the ICU from the ED [1–3], but strategies to decrease unplanned ICU transfers after ED admissions are still lacking. Many unplanned ICU transfers are due to sepsis. Delgado et al. found that respiratory tract infections, urinary tract infections, sepsis, and other acute infections are responsible for 26.9% of unplanned ICU transfers after ED admissions [4].

Many rules for stratifying the risk of ICU transfer in patients with sepsis have been advocated [5, 6]. However, it is unknown if the risk factors for unplanned ICU transfer in patients with infections are the same as those in patients without infections. Comparing the risk factors for unplanned ICU transfer in patients with infections and those without infections will enable the application of well-developed scoring systems used for sepsis to patients without infections. Our observational study was an attempt to make this comparison and to explore the feasibility of using risk stratification tools for sepsis to develop a predictive system for unplanned ICU transfer after ED admission.

## 2. Methods

### 2.1. Setting

This study was conducted in the ED of a suburban teaching hospital. Staffed by full-time emergency physicians (EPs), this ED has historically served approximately 50,000 patients annually with an admission rate of 25%, which accounts for 45% of the hospital's inpatients.

### 2.2. Study Design and Patients

**Figure 1:**
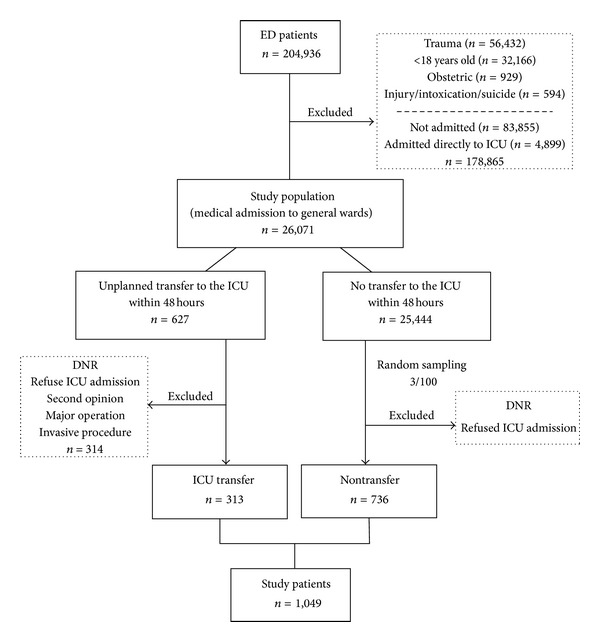
Development of ICU transfer group and nontransfer group.

Patients with nontraumatic conditions who underwent an unplanned transfer to the ICU within 48 hours of ED admission between January 1, 2007 and December 31, 2010 were included in the ICU transfer group. The control, or nontransfer group, included randomly selected patients who were not transferred to the ICU within 48 hours of admission. The ratio of controls to transfers was approximately 2 : 1 (736 controls, 313 transfers). If patients were to be admitted to a general ward but remained in the ED because of a delay or blocked access, they remained in the control group. Patients were excluded if they were younger than 18 years of age; were admitted for injuries, intoxications, suicides, or obstetric problems; had signed do not resuscitate (DNR) orders; or had critical conditions but initially refused ICU admission. Patients were also excluded if they showed no clinical deterioration after admission but were transferred to the ICU for a second opinion on their potential risk. Patients who were transferred to the ICU within 48 hours for close monitoring after a major operation or invasive procedure were not enrolled in the study, as these were considered expected transfers. The development of the study's 2 groups is illustrated in Figure 1. Patients with real clinical deterioration that led to unplanned ICU transfer were the focus of this study, so “deterioration” and “unplanned ICU transfer” are used interchangeably in the following text.

Two research nurses, each with at least 3 years of experience in emergency medicine or critical care, reviewed the medical records and abstracted the data on a structured data sheet. Another research assistant was responsible for data entry. One research nurse checked the data entry for accuracy. A board-certified EP confirmed the quality of the data and data sheets by establishing criteria for their logical validity. The research nurses were trained on the objective of the study, the definitions of the variables, and the techniques for reviewing medical records and abstracting data. Both electronic and written medical records were reviewed. The research nurses reviewed diagnoses given during outpatient visits and hospitalizations, medications used, and results of examinations to verify the presence of certain important comorbid illnesses.

### 2.3. Candidate Predictor Variables

The candidate predictor (independent) variables included specific pieces of data relating to demographics, comorbid conditions, chronic organ insufficiencies, physiological responses to disease, and organ dysfunctions. The comorbid conditions were partly drawn from the Charlson comorbidity index [7], and chronic organ insufficiencies were derived from Acute Physiology and Chronic Health Evaluation (APACHE) scores [8]. We used the standard definition of systemic inflammatory response syndrome (SIRS) [9] but set the thresholds for analyzing individual physiological response at a heart rate (HR) ≥130/min and a respiratory rate (RR) ≥30/min. These threshold values are the same as highest scores in the modified early warning score (MEWS) system commonly used in Europe [10, 11]. The definitions of organ dysfunctions were drawn from the criteria for severe sepsis [9], except that pulmonary dysfunction was defined as an pulse oximeter oxygen saturation (SpO_2_) at triage <90%, a lowest SpO_2_ <95% with use of oxygen, or a ratio of partial pressure of oxygen to fraction of inspired oxygen (PaO_2_/FiO_2_) <250 in an arterial blood gas analysis. Symptoms and signs used as calling criteria for a medical emergency team (MET) [12] were also recorded with some modifications: respiratory compromise was defined as an RR ≥30/min, the presence of moderate to severe respiratory distress efforts, or an SpO_2_ <90% with an increased respiratory rate (>20/min).

All infections from any organ system were categorized as “infections” in our study, except that meningitis and central nervous system infections were categorized as neurological diseases. Intra-abdominal diseases that had developed to peritonitis and/or presented with toxic signs of infection were also categorized as infections.

Vital signs and physiological changes were recorded at 4-time points: triage, decision of disposition, immediately before leaving the ED (or for those who deteriorated while in the ED, the last time these signs were recorded before deterioration), and time of clinical deterioration. The first 3-time points were grouped into the period “before deterioration.” Only those symptoms or signs that occurred before deterioration were used as candidate predictors of unplanned ICU transfer. Because there were no predetermined criteria for emergency physicians to decide if certain tests (e.g., arterial blood gases, liver enzymes, coagulation tests, or lactate levels) would be ordered, results of tests were considered to be negative in our study if they were not ordered.

### 2.4. Data Analysis

All variables with predefined thresholds were dichotomized, and univariate analyses were performed using Fisher's exact test. Patients with infections and those without infections were analyzed separately for risks for unplanned ICU transfer within 48 hours after ED admission. Those candidate predictors with a *P* value <0.1 were entered in the logistic regression analysis. Model discrimination was assessed by calculating the area under the receiver operating characteristic curve (AUC).

This study was approved by the Institutional Review Board of this institution and was exempt from obtaining patients' informed consent.

## 3. Results

Of the 204,936 ED visits in the study period, 26,071 patients with nontraumatic conditions were admitted to general wards and met our inclusion criteria. Of the 26,071 ED admissions, 627 underwent an unplanned ICU transfer within 48 hours; after excluding 314 of these patients based on our study criteria, 313 patients remained, comprising the ICU transfer group. Of the patients who were admitted to general wards but not transferred to the ICU within 48 hours, 736 were randomly selected, creating the nontransfer group (Figure 1). Of these 1049 patients, 605 (57.7%) were male, 492 (46.9%) were elderly (>65 years), and 202 (19.3%) were aged >80 years.

Three hundred and fifty (33.3%) of our study patients presented with infections. Patients with infections were more likely to have diabetes, cerebrovascular disease, dementia, or a previous history of respiratory failure and were more likely to be older, or in a cerebral performance category of 3 or 4. They were less likely to have coronary artery disease or a history of congestive heart failure compared to the patients without infections (Table 1).

The largest proportion of patients presented to the ED between 8:00 AM and 4:00 PM (492, 46.9%), followed by those presenting from 4:00 PM to 12:00 AM (367, 35.0%), and from 12:00 AM to 8:00 AM (190, 18.1%). The majority of our patients were discharged home (901, 85.9%), while 91 (8.7%) died as inpatients. Patients admitted for infections had a higher inpatient mortality rate compared to that for patients without infections (42 [12.0%] vs. 49 [7.0%], *P* = 0.01), and they were more likely than those without infections to have an unplanned ICU transfer within 48 hours after ED admission (135 [38.6%] vs. 178 [25.5%], *P* < 0.001). Patients with infections were more likely to deteriorate in the ED than in a general ward after transfer (56 [41.5%] vs. 52 [29.2%], *P* = 0.03). When deteriorations occurred, 38 patients (12.1%) needed cardiopulmonary resuscitation (CPR) and/or endotracheal intubations (Table 2).

The risk factors for unplanned ICU transfer in patients with infections were comparable to those in patients with other noninfectious diseases. A total of 24 variables were associated with unplanned ICU transfer in at least one of the study groups. These variables covered multiple dimensions of predisposition, physiological responses, organ dysfunctions, and other serious symptoms and signs listed in the calling criteria for an MET. Sixteen of these variables (66.7%) displayed this association in both study groups. These common risk factors included comorbidities (diabetes, coronary artery disease, a cerebral performance category of 3 or 4, and a previous history of congestive heart failure, severe liver disease, or end-stage renal disease), physiological responses (HR ≥ 130/min, abnormal white blood cells, and SIRS), organ dysfunctions (hypotension and pulmonary, renal, hematological, and metabolic dysfunctions), and symptoms or signs from the calling criteria for an MET (respiratory compromise and altered mental status). Having cerebrovascular disease or advanced malignancy or being immunocompromised or older than 65 years was associated with unplanned ICU transfer only in patients with infections, while a previous history of respiratory failure, liver dysfunction, seizure, or chest pain was associated with unplanned ICU transfer only in patients without infections (Table 3).

The independent risk factors for unplanned ICU transfer that occurred in patients with infections and those without infections were having a previous history of end-stage renal disease or presenting with SIRS, hypotension, renal dysfunction, hematological dysfunction, or altered mental status. Being older than 65 years or having a previous history of advanced malignancy independently predicted unplanned ICU transfer only in patients with infections. A previous history of severe liver disease or respiratory failure, a cerebral performance category of 3 or 4, a HR ≥130/min, and new-onset chest pain were independent predictors of unplanned ICU transfer only in patients without infections (Table 4). The AUC of the logistic regression model in patients with infections was 0.91 (95% CI, 0.87–0.96) and in patients without infections was 0.80 (95% CI, 0.76–0.84).

## 4. Discussion

Strategies aimed at recognizing patients at risk for deterioration and in need of critical care after ED admission may prevent unplanned ICU transfers and decrease the number of deaths in hospitals. However, few studies have compared the risk factors for unplanned ICU transfer in patients with infections and those without infections. In our study, these risk factors were shown to be comparable in both groups. Unplanned ICU transfer was found to be associated with 24 variables, either in patients with infections or those without infections, and two thirds of these variables were common to both groups. In addition, it was discovered that the risks for unplanned ICU transfer after ED admission, which included specific demographic data, comorbid illnesses, physiological changes, organ dysfunctions, and other serious symptoms and signs, could be organized according to the PIRO classification. In our unpublished study, a system was developed which successfully predicted unplanned ICU transfer after ED admission. The median PIRO score in that study was higher in the ICU transfer group than in the nontransfer group, and with a higher PIRO score, the proportions of patients with clinical deterioration increased. Inpatient mortality also partly increased with higher PIRO scores. These findings imply that it would be feasible to formally derive a system for predicting unplanned ICU transfer after ED admission using clinical variables organized according to a classification system such as PIRO.

Other researchers have previously attempted to assess the risk of unplanned ICU transfer after ED admission [13, 14]. Their studies, however, were based primarily on administrative data, which may not coincide with the decision-making behavior of EPs. Apart from these studies using administrative data, there have been few attempts to research this topic, and the efforts that have been made have not been integrated into a coherent body of results. On the other hand, risk factors for inpatient mortality after admission to general wards have been much more comprehensively studied, and some of the resulting insights may be transferable to the present topic.

Geriatric presentations to the ED are often atypical and involve multiple comorbidities [15], which not only complicate diagnosis and treatment, but also increase the risk of death. Goldhill and Sumner found that nonsurvivors of ICU admission were older than survivors [16]. In addition, the presence of comorbid illnesses increases the risk of death [17, 18]. History of organ insufficiency is widely used in scoring systems (the APACHE system being the most famous) to predict mortality in critically ill patients [19]. In this study, the risk factors of unplanned ICU transfer in patients with infections or those without infections included old age; a comorbidity of diabetes, coronary artery disease, or cerebral vascular disease; a cerebral performance category of 3 or 4; and a previous history of respiratory failure, congestive heart failure, severe liver disease, end-stage renal disease, advanced malignancy, or immune system-compromising conditions. However, only end-stage renal disease and advanced malignancy were identified as independent predictors of unplanned ICU transfer in patients with infections, and a history of end-stage renal disease, severe liver disease, or respiratory failure and a cerebral performance category of 3 or 4 independently predicted unplanned ICU transfer in patients without infections.

In addition to the demographic features and comorbidities discussed above, physiological changes such as tachypnea and shock are associated with unplanned ICU transfer [20, 21]. The modified early warning score (MEWS) aggregates such changes into a single-parameter weighted score, taking into account systolic blood pressure, heart rate, respiratory rate, body temperature, and neurological status [10]. This MEWS system, initially used to recognize deterioration in ward patients, has been validated as a predictor of inpatient mortality and the need for ICU admission in ED patients [10, 11, 22, 23].

Patients with severe sepsis have higher mortality rates [24], but there is little research on the impact of individual organ dysfunction on mortality. Fischer et al. found that abnormal coagulation tests are associated with mortality in ED patients with suspected infection [25], while in patients with severe sepsis, mortality is associated with elevated serum lactate levels [26]. In our study, patients with infections and organ dysfunction (hypotension or pulmonary, renal, liver, hematological, or metabolic dysfunction), who by definition had severe sepsis, were more likely to have an unplanned ICU transfer. These organ dysfunctions (except liver dysfunction) also predicted unplanned ICU transfer in patients without infections. In addition, hypotension, renal dysfunction, and hematological dysfunction seemed to be common pathways to deteriorations, since they independently predicted unplanned ICU transfer in patients with infections and those without infections.

In the literature, risk assessments for deterioration or inpatient mortality in undifferentiated ED patients are similar to those in patients with infection or sepsis. Kennedy et al. found that independent predictors of ICU transfer in infected ED patients included respiratory compromise, congestive heart failure, peripheral vascular disease, systolic blood pressure <100 mm Hg, a heart rate >90/min, and a creatinine level ≥2.0 mg/dL [5]. Shapiro et al. identified several independent predictors of death in ED patients with sepsis, including older age, terminal illness, nursing home residency, lower respiratory infections, tachypnea or hypoxia, septic shock, altered mental status, elevated proportion of bands in the white blood cell count, and thrombocytopenia, and proceeded to propose a risk-prediction system called Mortality in Emergency Department Sepsis (MEDS) [6]. The MEDS system has been validated to be an effective tool for risk stratification in patients with severe sepsis [27, 28] and was further organized using the PIRO classification [29], as recommended by the International Sepsis Definition Conference in 2001 [30]. The PIRO model, with its multidimensional predictive variables, has been validated in risk staging for sepsis [31–33]. Kellett and Deane, while developing the Simple Clinical Score (SCS), identified 16 independent predictors of 30-day mortality after undifferentiated ED admissions [34]. These variables, similar to those grouped under the PIRO concept, included age, diabetes, nursing home residency, prior chronic conditions limiting daily activities, neurological presentations, cardiac presentations, and vital sign changes. A summary of all variables that were predictive of unplanned ICU transfer or inpatient mortality in our study and the broader literature is presented in Table 5.

Because our study was retrospective, observational, and conducted at a single institution, there are some limits to the generalization of its results. Very simply, patients with clinical deteriorations that led to unplanned ICU transfers were the focus of the study, in the hopes that our results would be applicable to other institutions with different resources and admission policies. The chart reviews had flaws common to such methodologies, involving some inaccuracy and incompleteness in vital sign measurements and the recording of medical events, and inconsistent criteria for ordering certain examinations and identifying abnormalities during these examinations. However, vital signs and laboratory results were included in our risk evaluations, contributing to the existing literature for the evaluation of the risk of unplanned ICU transfer after ED admission. In addition, retrospective studies similar to ours help to preserve blindness when assessing outcomes, an important feature when one hopes to construct a clinically predictive model such as the one presented in our study [35].

## 5. Conclusions

The risks of unplanned ICU transfer in patients with infections were found to be comparable to those in patients without infections, and the risk factors for unplanned ICU transfer included variables from multiple dimensions that could be organized according to the PIRO classification system. It would be feasible to use such variables, organized by PIRO or a comparable system, to derive a predictive system for unplanned ICU transfer.

## Figures and Tables

**Table 1:** Demographics and comorbidities in patients with infections and those without infections.

	With infection *n* = 350	Without infection *n* = 699	Combined *N* = 1049	*P* ^a^
Sex				
Male	185 (52.9)	420 (60.1)	605 (57.7)	0.03
Age, mean (standard deviation)	62.3 (20.4)	59.4 (18.9)		0.03^b^
Age				0.01
18–44 years	74 (21.1)	179 (25.6)	253 (24.1)	
45–64 years	95 (27.1)	209 (29.9)	304 (29.0)	
65–79 years	94 (26.9)	196 (28.0)	290 (27.6)	
≥80 years	87 (24.9)	115 (16.5)	202 (19.3)	
Comorbidities				
Hypertension	151 (43.1)	316 (45.2)	467 (44.5)	0.53
Diabetes	133 (38.0)	194 (27.8)	327 (31.2)	0.001
Cerebrovascular disease	78 (22.3)	111 (15.9)	189 (18.0)	0.01
Coronary artery disease	40 (11.4)	127 (18.2)	167 (15.9)	0.005
Alcohol abuse	26 (7.4)	67 (9.6)	93 (8.9)	0.25
Dementia	40 (11.4)	33 (4.7)	73 (7.0)	<0.001
Cerebral performance category of 3 or 4	84 (24.0)	56 (8.0)	140 (13.3)	<0.001
Histories of organ failure				
Respiratory failure	20 (5.7)	20 (2.9)	40 (3.8)	0.02
Congestive heart failure	31 (8.9)	96 (13.7)	127 (12.1)	0.02
Severe liver disease	19 (5.4)	51 (7.3)	70 (6.7)	0.25
End-stage renal disease	17 (4.9)	29 (4.1)	46 (4.4)	0.60
Malignancy, advanced	34 (9.7)	62 (8.9)	96 (9.2)	0.66
Immunocompromised	11 (3.1)	12 (1.7)	23 (2.2)	0.14

Data are expressed as *n* (%) unless otherwise noted.

^a^Chi-square.

^b^Student's *t* test.

**Table 2:** Clinical features and outcomes in patients with infections and those without infections.

	With infections n = 350	Without infections n = 699	Combined *N* = 1049	P^a^
Times of arrival				0.10
12 AM to 8 AM	61 (17.4)	129 (18.5)	190 (18.1)	
8 AM to 4 PM	151 (43.1)	341 (48.8)	492 (46.9)	
4 PM to 12 AM	138 (39.4)	229 (32.8)	367 (35.0)	
Outcomes				0.003
Discharged home	282 (80.6)	619 (88.6)	901 (85.9%)	
Death	42 (12.0)	49 (7.0)	91 (8.7%)	0.01^b^
Transfer to chronic care facility	24 (6.9)	25 (3.6)	49 (4.7%)	
Transfer to another hospital	2 (0.6)	6 (0.9)	8 (0.8%)	
Deterioration within 48 hours (Nd)	135 (38.6)	178 (25.5)	313 (29.8)	<0.001^b^
Site when deteriorations occurred *n* (% within Nd)				0.03^b^
Emergency department	56 (41.5)	52 (29.2)	108 (34.5)	
Ward	79 (58.5)	126 (70.8)	205 (65.5)	
Resuscitation upon deterioration *n* (% within Nd)				0.89
No resuscitation	119 (88.1)	156 (87.6)	275 (87.9)	
CPR^c^-death	1 (0.7)	3 (1.7)	4 (1.3)	
CPR-ROSC^d^	6 (4.4)	6 (4.4)	13 (4.2)	
Endotracheal intubation only	9 (6.7)	9 (6.8)	21 (6.7)	

Data are expressed as *n* (%) unless otherwise noted.

^a^Chi-square.

^b^Fisher's exact.

^c^CPR: cardiopulmonary resuscitation.

^d^ROSC: recovery of spontaneous circulation.

**Table 3:** Risk of deterioration in patients with infections and those without infections.

Candidate variables	With infections				Without infections			
	ICU transfer n = 135	Non-transfer n = 215	OR (95% CI)	P^a^	ICU transfer n = 178	Non-transfer n = 521	OR (95% CI)	P^a^
Demographics and comorbidities								
≥65 years old	87 (64.4)	94 (43.7)	2.3 (1.5–3.6)	<0.001	88 (49.4)	223 (42.8)	1.3 (0.9–1.8)	0.14
Diabetes	63 (46.7)	70 (32.6)	1.8 (1.2–2.8)	0.009	66 (37.1)	128 (24.6)	1.8 (1.3–2.6)	0.002
Coronary artery disease	22 (16.3)	18 (8.4)	2.1 (1.1–4.1)	0.03	43 (24.2)	84 (16.1)	1.7 (1.1–2.5)	0.02
Cerebrovascular disease	39 (28.9)	39 (18.1)	1.8 (1.1–3.0)	0.03	36 (20.2)	75 (14.4)	1.5 (1.0–2.3)	0.08
Cerebral performance category of 3 or 4	49 (36.3)	35 (16.3)	2.9 (1.8–4.9)	<0.001	28 (15.7)	28 (5.4)	3.3 (1.9–5.7)	<0.001
Histories of organ failure								
Respiratory failure	10 (7.4)	10 (4.7)	1.6 (0.7–4.1)	0.35	14 (7.9)	6 (1.2)	7.3 (2.8–19.4)	<0.001
Congestive heart failure	20 (14.8)	11 (5.1)	3.2 (1.5–7.0)	0.003	38 (21.3)	58 (11.1)	2.2 (1.4–3.4)	0.001
Severe liver disease	12 (8.9)	7 (3.3)	2.9 (1.1–7.6)	0.03	29 (16.3)	22 (4.2)	4.4 (2.5–7.9)	<0.001
End-stage renal disease	11 (8.1)	6 (2.8)	3.1 (1.1–8.6)	0.04	13 (7.3)	16 (3.1)	2.5 (1.2–5.3)	0.03
Malignancy, advanced	26 (19.3)	8 (3.7)	6.2 (2.7–14.1)	<0.001	18 (10.1)	44 (8.4)	1.2 (0.7–2.2)	0.54
Immunocompromised	8 (5.9)	3 (1.4)	4.5 (1.2–17.1)	0.03	5 (2.8)	7 (1.3)	2.1 (0.7–6.8)	0.19
Physiological responses								
HR ≥ 130/min	24 (17.8)	18 (8.4)	2.4 (1.2–4.6)	0.01	53 (29.8)	110 (21.1)	1.6 (1.1–2.3)	0.03
Abnormal white blood cell^b^	77 (57.0)	93 (43.3)	1.7 (1.1–2.7)	0.02	24 (13.5)	20 (3.8)	3.9 (2.1–7.3)	<0.001
SIRS^c^	100 (74.1)	104 (48.4)	3.0 (1.9–4.9)	<0.001	88 (49.4)	103 (19.8)	4.0 (2.8–5.7)	<0.001
Organ dysfunctions								
Hypotension	32 (23.7)	10 (4.7)	6.4 (3.0–13.4)	<0.001	20 (11.2)	16 (3.1)	4.0 (2.0–7.9)	<0.001
Pulmonary dysfunction	35 (25.9)	18 (8.4)	3.8 (2.1–7.1)	<0.001	25 (14.0)	19 (3.6)	4.3 (2.3–8.0)	<0.001
Renal dysfunction	32 (23.7)	5 (2.3)	13.0 (4.9–34.4)	<0.001	29 (16.3)	41 (7.9)	2.3 (1.4–3.8)	0.002
Liver dysfunction	7 (5.2)	2 (0.9)	5.8 (1.2–28.4)	0.02	6 (3.4)	7 (1.3)	2.6 (0.8–7.7)	0.11
Hematological dysfunction	21 (15.6)	7 (3.3)	5.5 (2.3–13.3)	<0.001	26 (14.6)	23 (4.4)	3.7 (2.1–6.7)	<0.001
Metabolic dysfunction	12 (8.9)	3 (1.4)	6.9 (1.9–24.9)	0.002	13 (7.3)	5 (1.0)	8.1 (2.9–23.1)	<0.001
Symptoms/signs from MET^d^ criteria								
Respiratory compromise	35 (25.9)	16 (7.4)	4.4 (2.3–8.2)	<0.001	26 (14.6)	19 (3.6)	4.5 (2.4–8.4)	<0.001
Altered mental status	5 (3.7)	1 (0.5)	8.2 (1.0–71.2)	0.03	19 (10.7)	2 (0.4)	31 (7.1–134.6)	<0.001
Seizure	1 (0.7)	0 (0)	NA^e^	0.39	10 (5.6)	10 (1.9)	3.0 (1.2–7.4)	0.02
Chest pain (new onset)	0 (0)	0 (0)	NA^e^	NA^e^	10 (5.6)	7 (1.3)	4.4 (1.6–11.7)	0.003

Data are expressed as *n* (%) unless otherwise noted.

^a^Fisher's exact test.

^b^White blood cell count of >12,000/µL or <4000/µL or bands >5%.

^c^Systemic inflammatory response syndrome.

^d^Medical emergency team.

^e^Not applicable.

**Table 4:** Multiple logistic regression analyses for risk of unplanned ICU transfer.

AUC^a^ (95% CI)	With infections		Without infections	
	0.91 (0.87–0.96)		0.80 (0.76–0.84)	
Variable	OR (95% CI)	P^b^	OR (95% CI)	P^b^
Predispositions				
End-stage renal disease	4.0 (1.2–12.8)	0.02	3.1 (1.3–7.2)	0.009
≥65 years old	2.8 (1.6–4.9)	<0.001		
History of advanced malignancy	5.2 (2.0–13.3)	0.001		
Severe liver disease			4.1 (2.0–8.4)	<0.001
History of respiratory failure			4.4 (1.5–12.7)	0.007
Cerebral performance category of 3 or 4			2.9 (1.5–5.6)	0.001
Responses				
SIRS^c^	3.1 (1.8–5.5)	<0.001	2.7 (1.7–4.2)	<0.001
HR ≥ 130/min			3.0 (1.4–6.1)	0.003
Organ dysfunctions				
Hypotension	4.0 (1.7–9.5)	0.002	2.8 (1.3–5.9)	0.009
Renal dysfunction	10.6 (3.8–29.9)	<0.001	2.0 (1.1–3.6)	0.03
Hematological dysfunction	5.9 (2.1–16.6)	0.001	2.6 (1.2–5.3)	0.01
Symptoms/signs from MET^d^ criteria				
Altered mental status	12.1 (1.0–142.2)	0.048	34.5 (7.5–158.3)	<0.001
New-onset chest pain			9.4 (3.3–27.1)	<0.001

^a^Area under receiver characteristic curve.

^b^Fisher's exact test.

^c^Systemic inflammatory response syndrome.

**Table 5:** Summary of variables predictive of deterioration/mortality in the literature and present study.

Risk predictor/outcome variable	Kennedy et al.^a^	Shapiro et al.^b^	Kellett and Deane^c^	With infections^ d^	Without infections^ d^
	ICU transfer	Mortality	Mortality	ICU transfer	ICU transfer
Demographics and comorbidities				(*P*)	(*P*)
≥65 years old		●	●	<0.001	NS^e^
Diabetes			●	0.009	0.002
Coronary artery disease				0.026	0.018
Cerebrovascular disease				0.025	NS^e^
Peripheral vascular disease	●				
Cerebral performance category of 3 or 4/nursing home resident		●	●	<0.001	<0.001
Histories of organ failure					
Respiratory failure	●			NS^e^	<0.001
Congestive heart failure	●			0.003	0.001
Severe liver disease				0.029	<0.001
End-stage renal disease				0.038	0.027
Malignancy, advanced		●		<0.001	NS^e^
Immunocompromised				0.026	NS^e^
Insult (lower respiratory tract infection)		●			
Physiological response					
Tachycardia	●		●	0.011	0.024
Abnormal white blood cell		●		0.016	<0.001
Abnormal body temperature			●		
SIRS^f^				<0.001	<0.001
Organ dysfunctions					
Hypotension	●	●	●	<0.001	<0.001
Pulmonary dysfunction/hypoxia		●	●	<0.001	<0.001
Renal dysfunction	●			<0.001	0.002
Liver dysfunction				0.019	NS^e^
Hematological dysfunction		●		<0.001	<0.001
Metabolic dysfunction				0.002	<0.001
Symptoms/signs from MET criteria					
Respiratory compromise			●	<0.001	<0.001
Altered mental status		●	●	0.034	<0.001
Seizure				NS^e^	0.017
New-onset chest pain			●	NA^g^	0.003

Data are expressed as *n* (%) unless otherwise noted.

^a^Infection patients [5].

^b^Infection patients [6].

^c^Undifferentiated patients [34].

^d^The present study.

^e^Non-significant.

^f^Systemic inflammatory response syndrome.

^g^Not applicable.
